# Automated T2 relaxometry of the hippocampus for temporal lobe epilepsy

**DOI:** 10.1111/epi.13843

**Published:** 2017-07-12

**Authors:** Gavin P. Winston, Sjoerd B. Vos, Jane L. Burdett, M. Jorge Cardoso, Sebastien Ourselin, John S. Duncan

**Affiliations:** ^1^ Department of Clinical and Experimental Epilepsy UCL Institute of Neurology London United Kingdom; ^2^ Epilepsy Society MRI Unit Chalfont St Peter United Kingdom; ^3^ Translational Imaging Group Centre for Medical Image Computing UCL London United Kingdom

**Keywords:** Hippocampus, T2 relaxometry, Temporal lobe epilepsy, Magnetic resonance imaging

## Abstract

**Objective:**

Hippocampal sclerosis (HS), the most common cause of refractory temporal lobe epilepsy, is associated with hippocampal volume loss and increased T2 signal. These can be identified on quantitative imaging with hippocampal volumetry and T2 relaxometry. Although hippocampal segmentation for volumetry has been automated, T2 relaxometry currently involves subjective and time‐consuming manual delineation of regions of interest. In this work, we develop and validate an automated technique for hippocampal T2 relaxometry.

**Methods:**

Fifty patients with unilateral or bilateral HS and 50 healthy controls underwent T_1_‐weighted and dual‐echo fast recovery fast spin echo scans. Hippocampi were automatically segmented using a multi‐atlas–based segmentation algorithm (STEPS) and a template database. Voxelwise T2 maps were determined using a monoexponential fit. The hippocampal segmentations were registered to the T2 maps and eroded to reduce partial volume effect. Voxels with T2 >170 msec excluded to minimize cerebrospinal fluid (CSF) contamination. Manual determination of T2 values was performed twice in each subject. Twenty controls underwent repeat scans to assess interscan reproducibility.

**Results:**

Hippocampal T2 values were reliably determined using the automated method. There was a significant ipsilateral increase in T2 values in HS (p < 0.001), and a smaller but significant contralateral increase. The combination of hippocampal volumes and T2 values separated the groups well. There was a strong correlation between automated and manual methods for hippocampal T2 measurement (0.917 left, 0.896 right, both p < 0.001). Interscan reproducibility was superior for automated compared to manual measurements.

**Significance:**

Automated hippocampal segmentation can be reliably extended to the determination of hippocampal T2 values, and a combination of hippocampal volumes and T2 values can separate subjects with HS from healthy controls. There is good agreement with manual measurements, and the technique is more reproducible on repeat scans than manual measurement. This protocol can be readily introduced into a clinical workflow for the assessment of patients with focal epilepsy.

**Key Points**

- Hippocampal sclerosis (HS) is characterized by volume loss and increased T2 signal
- Quantitative imaging with hippocampal volumetry and T2 relaxometry improves sensitivity in detecting HS
- Hippocampal T2 values can be reliably determined using an automated technique, rather than time‐consuming manual delineation
- Automated measurement shows an interscan reproducibility superior to that with manual measurement
- The combination of hippocampal volumes and hippocampal T2 values can reliably identify sclerotic hippocampi

The most common cause of medically refractory temporal lobe epilepsy (TLE) is hippocampal sclerosis (HS) characterized by neuronal cell loss and gliosis within the hippocampus, particularly CA1, CA3, and the dentate gyrus.^1^ Surgical treatment is often required to achieve seizure freedom, and invasive investigations may be avoided if an abnormality can be detected on magnetic resonance imaging (MRI) that is concordant with other clinical data including semiology, video–electroencephalography (EEG) telemetry and neuropsychology. Identification of a focal abnormality is associated with a better surgical outcome.^2^

Typical MRI features of HS include hippocampal atrophy, disrupted internal hippocampal structure, and decreased T_1_‐weighted and increased T_2_‐weighted signal.^3^ Because visual analysis alone may fail to detect HS,^4^ quantitative imaging including hippocampal volumetry and T2 relaxometry can improve sensitivity.^5^, ^6^, ^7^

Hippocampal volumetry can quantify volume loss (atrophy), representing neuronal cell loss that may be difficult to appreciate visually.^8^, ^9^ Atrophy correlates with side of seizure onset^10^ and postoperative outcome after anterior temporal lobe resection.^11^ Although volumetry was initially based on time‐consuming manual delineation of the hippocampi, we and other groups have developed automated techniques to determine hippocampal volumes.^12^

T2 relaxometry measures T2 relaxation time, an intrinsic tissue property. It may detect subtle pathology, which is useful in lateralizing temporal lobe epilepsy, even in the absence of hippocampal atrophy,^5^, ^6^ and has been reported to be the most consistent imaging finding in HS.^13^ T2 maps were initially generated by lengthy multiecho acquisitions such as a Carr‐Purcell‐Meiboom‐Gill sequence.^5^ However, rapid whole brain coverage can be reliably obtained with a dual‐echo spin‐echo sequence,^14^, ^15^, ^16^ with T2 values determined by fitting a monoexponential to the two values.

The current clinical approach to hippocampal T2 measurement involves manual delineation of circular/elliptical regions of interest (ROIs).^5^, ^8^, ^14^, ^16^ This is time consuming, and difficulties may arise in the suitable placement of a region of interest while avoiding contamination from cerebrospinal fluid (CSF). In this study, we seek to automate and validate an automated method for T2 relaxometry of the hippocampus that addresses these concerns.

## Methods

### Subjects

We selected 50 patients who had undergone brain MRI with an epilepsy protocol as part of routine clinical practice for TLE at the Epilepsy Society MRI Unit, Chalfont St Peter, and which been reported by a neuroradiologist as showing unilateral or bilateral HS on visual assessment (median age 40 years, range 18–76, 23 male). We recruited 50 healthy controls (median age 37 years, range 17–66, 28 male) without any history of neurologic or psychiatric disease.

The study was considered a service evaluation using clinically acquired data by the National Hospital for Neurology and Neurosurgery and the Institute of Neurology Joint Research Ethics Committee. Informed written consent was obtained from control subjects.

### Data acquisition

Subjects underwent imaging on a 3T GE MR750 scanner with a 32‐channel coil. Sequences included a three‐dimensional (3D) T_1_‐weighted inversion‐recovery fast spoiled gradient recalled echo (TE/TR/TI 3.1/7.4/400 msec, field of view (FOV) 224 × 256 × 256 mm, matrix 224 × 256 × 256, parallel imaging acceleration factor 2) and a coronal dual‐echo fast recovery fast spin echo proton‐density/T_2_‐weighted (TE 30/119 msec, TR 7,600 msec, FOV 220 × 220 mm, matrix 512 × 512, slice thickness 4 mm, SENSE factor 2).

### Data processing

**Figure 1:**
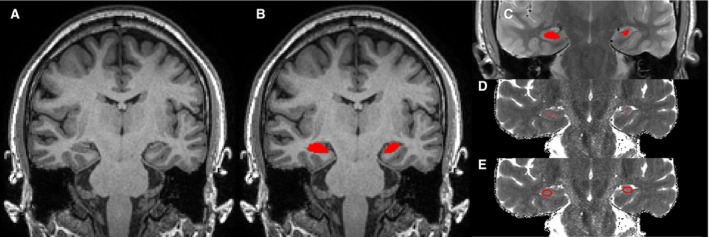
Example of a patient with left hippocampal sclerosis. Structural T_1_‐weighted scan (**A**) with automated hippocampal segmentation (**B**). The segmentation is registered to the dual‐echo PD/T_2_‐weighted scan, eroded, and CSF excluded (**C**), and the mean T2 value determined on the voxelwise T2 map (**D**). Manual determination of T2 values relies of manually drawn elliptic ROIs (**E**).

Hippocampi were automatically segmented from the T_1_‐weighted volume using a multi‐atlas–based segmentation algorithm (STEPS) and a template database of 400 manual segmentations as previously described (Fig. 1A,B)^12^ and widely available online (https://hipposeg.cs.ucl.ac.uk/). Hippocampal volumes were corrected for intracranial volume using linear regression with parameters derived from a group of healthy controls.

Voxelwise T2 maps were determined using a monoexponential fit^14^ from the signal S_1_, S_2_ at the two echo times TE_1_, TE_2_:

$$T2\;=\;\frac{TE_{2}-TE_{1}}{\ln\left(\frac{S_{1}}{S_{2}}\right)}$$

A rigid transformation from the T_1_‐weighted image to the dual‐echo PD/T2 image was calculated using NiftyReg (https://sourceforge.net/projects/niftyreg) and applied to the hippocampal segmentations. The segmentations were eroded and voxels with T2 values >170 msec were eliminated to minimize cerebrospinal fluid (CSF) contamination (Fig. 1C), and the mean T2 value in this region of interest was determined (Fig. 1D). The value of 170 msec was determined empirically by segmenting the T_1_‐weighted images into gray matter, white matter, and CSF using New Segment in SPM8 (http://ww.fil.ion.ucl.ac.uk/spm), applying the above rigid transformation and measuring T2 values in the control subjects in each compartment. The distributions were reviewed to determine the optimal cutoff between the upper tail of T2 values in gray matter and the lower tail in CSF.

A script for determining hippocampal T2 values using the hippocampal segmentations from a T_1_‐weighted image obtained from our online hippocampal segmentation website (https://hipposeg.cs.ucl.ac.uk) and a locally calculated T2 map is available at https://github.com/sjoerdvos/hippocampal_T2.

### Comparison to manual determination

For comparison to previous practice, a radiographer manually determined hippocampal T2 values using elliptical ROIs on consecutive coronal slices (Fig. 1E), and the mean T2 across all ROIs was determined.^17^ This was performed in all subjects on two separate occasions to assess intrarater reproducibility.

### Interscan reproducibility

To compare interscan reproducibility of automated and manual measurement, 20 controls underwent repeat imaging on a separate occasion with the same protocol. Hippocampal T2 measurements were performed on the repeat scan with the automated and manual methods.

### Statistics

All statistical analysis was performed in IBM SPSS Statistics 24. Hippocampal volumes and hippocampal T2 values were normally distributed in each group according to the Shapiro‐Wilks test. Statistical comparison between groups was therefore made using a two‐tailed independent samples *t*‐test assuming equal variances. Gender and laterality effects in healthy controls were determined with two‐tailed independent samples and paired *t*‐tests, respectively.

Binary logistic regression was used to assess the classification of left and right hippocampi separately into pathologic or nonpathologic using the corresponding hippocampal volumes and hippocampal T2 values, both independently and combined.

Agreement between the automated and manual methods for determining hippocampal T2 was determined by correlation, as although the general distribution of values should be similar, exact agreement between the methods would not be expected due to the different sampling.

The reproducibility of T2 measurements between repeat scans in the same subject was assessed using Bland‐Altmann plots and a paired *t*‐test to check for equality of means.

## Results

### Hippocampal volumes and T2 by subgroup

**Figure 2:**
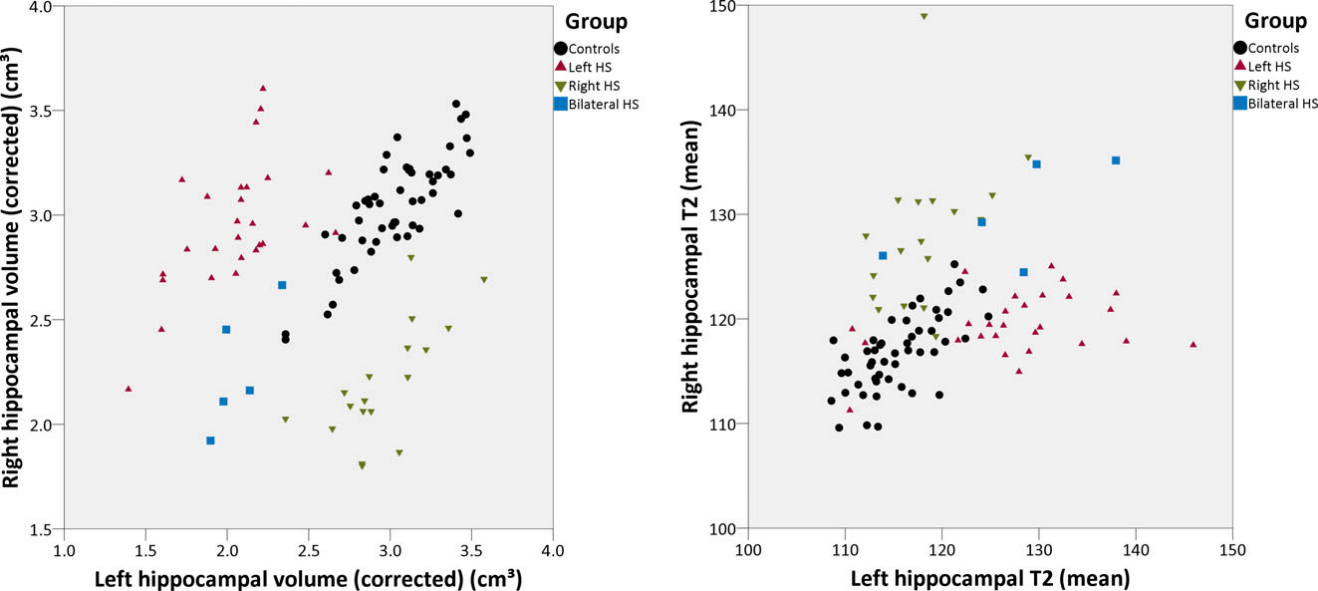
Scatterplot of hippocampal volumes corrected for intracranial volume (left) and mean hippocampal T2 (right) of both hippocampi obtained by the automated method in the different groups.

**Figure 3:**
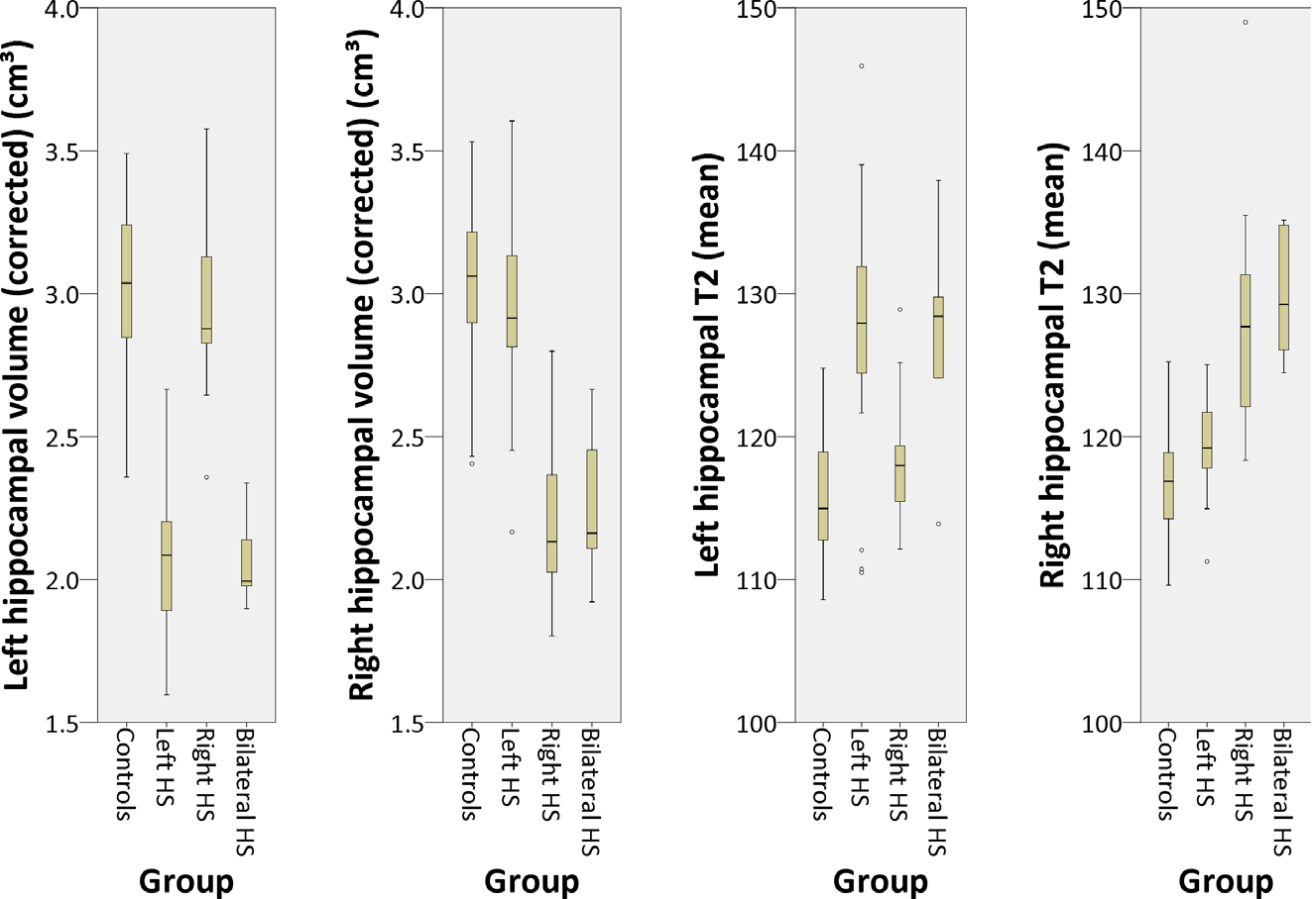
Box plots of hippocampal volumes corrected for intracranial volume and mean hippocampal T2 obtained by the automated method in the different groups.

Hippocampal volumes and T2 values were reliably determined using the automated method (Fig. 2, Table 1), with good separation between the groups on either hippocampal volumes or T2 values (Fig. 3).

**Table 1:** Hippocampal volumes corrected for intracranial volume and automated T2 values for each group given as mean (SD)

	Controls (n = 50)	Left HS (n = 27)	Right HS (n = 18)	Bilateral HS (n = 5)
L hippocampal volume	3.02 (0.28)	2.05 (0.30) [p < 0.001]	2.96 (0.28) [NS]	2.07 (0.17) [p < 0.001)
R hippocampal volume	3.04 (0.25)	2.95 (0.30) [NS]	2.20 (0.28) [p < 0.001]	2.26 (0.29) [p < 0.001]
Left hippocampal T2	115.5 (4.11)	127.7 (8.18) [p < 0.001]	118.1 (4.49) [p = 0.026]	126.8 (8.80) [p < 0.001]
Right hippocampal T2	116.8 (3.60)	119.5 (3.00) [p = 0.002]	128.1 (7.05) [p < 0.001]	129.9 (4.91) [p < 0.001]

p‐Values for a two‐tailed *t*‐test comparison to the control group are also given. NS, not significant.

Left hippocampal volumes were significantly reduced and left hippocampal T2 significantly increased in both left HS (two‐tailed *t*‐test, p < 0.001 for both) and bilateral HS (p < 0.001 for both) compared to controls. Right hippocampal volumes were significantly reduced and right hippocampal T2 significantly increased in right HS (p < 0.001 for both) and bilateral HS (p < 0.001 for both).

There were no contralateral changes in hippocampal volume, but mean right hippocampal T2 was mildly increased by 2.6 msec in left HS (p = 0.002) and mean left hippocampal T2 mildly increased by 2.7 msec in right HS (p = 0.026).

### Gender and laterality effects

There were no significant differences between male and female controls in hippocampal volumes or hippocampal T2 values. There were no laterality effects for hippocampal volume, but mean hippocampal T2 values were significantly lower on the left than the right, with both automated (115.5 msec versus 116.8 msec, p = 0.006) and manual (p = 0.034) measurements.

Reference ranges for hippocampal volumes and hippocampal T2 values determined from the healthy controls as mean ± 1.96 standard deviations (SDs) were 2.51–3.55 cm^3^ and 108.5–123.8 msec, respectively. Although this combines both sides, the relevant upper end of the reference range for hippocampal T2 was indistinguishable between the left (123.6 msec) and right (123.9 msec) hippocampi individually.

### Classification of hippocampi

**Figure 4:**
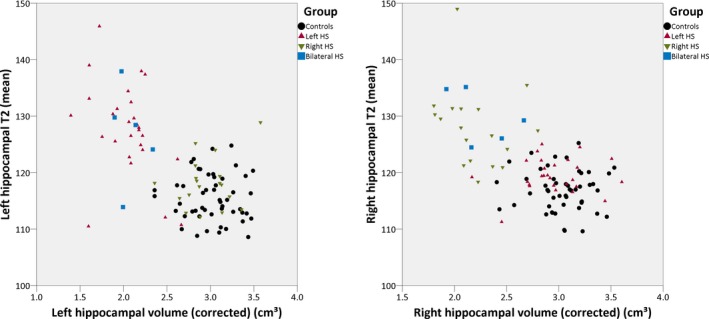
Scatter plot of hippocampal volumes corrected for intracranial volume and mean hippocampal T2 of the left and right hippocampi, respectively, showing good separation between the different groups.

The combination of hippocampal volume and T2 values gave good visual separation between groups (Fig. 4). Binary logistic regression could correctly classify 94% of left and 97% of right hippocampi as pathologic or nonpathologic with the neuroradiologic report forming the gold standard.

### Comparison to manual determination

**Figure 5:**
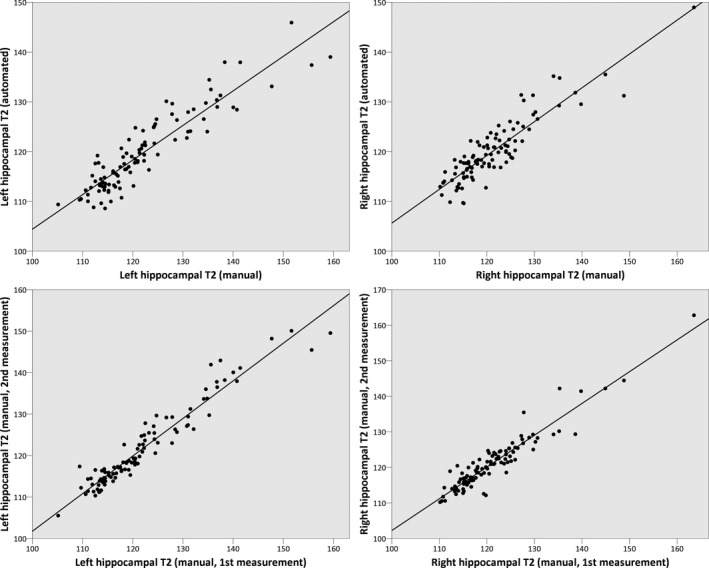
Comparison of automated and manual hippocampal T2 determination (top) and of intrarater reliability of manual determination on the same scans (bottom).

There was a strong correlation between the automated and manual methods for determining hippocampal T2 (Fig. 5) with Pearson correlations of 0.917 (left, p < 0.001) and 0.896 (right, p < 0.001). For comparison, the correlation between two manual measurements was 0.964 (left, p < 0.001) and 0.942 (right, p < 0.001).

### Interscan reproducibility

Bland‐Altman plots confirmed that hippocampal T2 measurements were reproducible between repeat scans. The pairwise differences in hippocampal T2 were not significantly different from zero for either automated or manual measurements. However, the standard deviation of the differences was greater for manual (6.00 msec) than for automated (3.59 msec) measurement, leading to wider intervals on the Bland‐Altman plot.

## Discussion

### Key findings

Automated hippocampal segmentation can be reliably extended to the determination of hippocampal T2 values, and a combination of hippocampal volumes and T2 values can separate subjects with HS from healthy controls. We found good agreement with manual measurements and an improved reproducibility on repeat scans compared to manual measurements.

### Role of T2 relaxometry

T_2_‐weighted hyperintensity, one of the key radiologic features of HS, can be objectively assessed by quantitative measurement of T2 relaxation (T2 mapping) with increased sensitivity over visual reading.^18^, ^19^ T2 values may be raised in the absence of atrophy^6^ and the combination of hippocampal atrophy with a high T2 value is both sensitive and specific for HS.^20^ Employing a combination of hippocampal volumes and T2 values can increase the yield to 99% of visually detected HS but also 28% of those considered visually normal.^7^

### Acquisition protocol

The initial scan protocols comprising multi‐echo Carr‐Purcell‐Meiboom‐Gill sequences were time‐consuming and gave limited spatial coverage.^5^ The more efficient dual‐echo approach has been validated^6^, ^14^, ^15^ despite its assumption of monoexponential decay in voxels with mixed tissue components and potential fitting errors from only two data points. Although multiecho approaches remain the accepted gold standard, dual‐echo approaches allow full brain coverage in a clinically feasible timescale with sufficient sensitivity and accuracy to detect pathologic changes.

Standard T_2_‐weighted clinical acquisitions may be analyzed^21^ but are less sensitive than T2 relaxometry.^22^ Computational analysis of conventional fluid‐attenuated inversion recovery (FLAIR) images is also less sensitive and specific than T2 relaxometry.^23^

### Method for determining hippocampal T2

The established approach of manual placement of circular/elliptical ROIs is time‐consuming and the regions are necessarily limited in size to around 20 mm^2^ per slice to avoid CSF contamination so only sample a small part of the hippocampus. By using an automated segmentation, we could determine the mean T2 value in the entire hippocampus where cross‐sectional areas in a slice can exceed 50 mm^2^. CSF contamination was minimized by eroding the segmentation at the boundary and employing a threshold derived empirically from T2 values in gray matter and CSF of healthy controls.

### Reliability and reproducibility

Manual determination of hippocampal T2 values has been shown to be reproducible over time and unaffected by single seizures.^24^ Here we show that automated measurements have superior reproducibility with repeat scans, which may reflect the sampling of the whole hippocampus rather than manually placed ROIs, avoiding CSF whose location may vary depending on acquisition orientation.

There is a good correlation between manual and automated measurements, but the values are not in exact agreement with the slope of regression lines being below unity (Fig. 5). This is not unexpected given the different sampling techniques but may arise from inclusion of voxels within the manually placed ROIs that have small CSF contributions. However, it highlights that any comparison of T2 values needs to use the same technique and acquisition protocol.^14^

### Pathologic correlates

HS is characterized histologically by cell loss and astrogliosis throughout mesial temporal limbic areas, especially the hippocampus.^1^ Although atrophy reflects cell loss and gliosis in CA1–3 and the dentate,^9^, ^25^ increased T2 values have been shown to correlate with dentate gliosis,^9^ neuronal cell loss in CA1 and CA3^16^ and gliosis and loss or dispersion of the granular cell layer.^26^

### Bilateral changes

A notable finding is the bilateral changes observed in hippocampal T2 values even with apparently unilateral pathology. This has been observed in histopathologic studies^1^ and previous imaging studies,^6^, ^16^, ^19^ with greater changes observed ipsilaterally. Possible reasons include bilateral hippocampal pathology with gliosis, mild edema from seizures, or drug effects.^5^ The ipsilateral hippocampal T2 changes observed in extratemporal lobe epilepsy lend support to seizure‐related changes.^27^ Contralateral changes are related to memory performance^28^ and poorer postoperative seizure outcome.^16^

### T2 relaxometry outside the hippocampus

Our approach has been limited to hippocampal T2, but structural damage extends beyond the hippocampus.^29^ ROI studies have shown T2 signal changes in temporal lobe white matter, amygdala, and frontal and parietal lobes.^30^, ^31^ Our technique could easily be extended to other structures delineated on the T_1_‐weighted image, but an alternative approach is whole‐brain voxel‐based relaxometry. This identifies changes in the temporal lobe white matter, amygdala, and parahippocampal gyrus,^32^, ^33^ and may be more sensitive than ROI‐based approaches.^34^

### Future work

Patients in this study were selected according to visually apparent HS. Future work will include those with normal‐appearing hippocampi, but histologically identified HS following surgical treatment and the automated T2 mapping protocol will be introduced into our standard clinical workflow.

We report a single value for each hippocampus representing the mean T2. As T2 values overlap between healthy controls and patients, it is critical that these single values are appropriately interpreted in a clinical setting in conjunction with hippocampal volumes, the clinical picture, and other available data.

Hippocampal T2 values are higher anteriorly than posteriorly in healthy controls, and in HS, elevation of T2 values may occur diffusely or predominantly anteriorly.^4^ A single figure may obscure subtle changes, and extending to a slice‐by‐slice profile of T2 values in an anteroposterior axis may provide useful additional information.

## Disclosure

None of the authors has any conflict of interest to disclose. We confirm that we have read the Journal's position on issues involved in ethical publication and affirm that this report is consistent with those guidelines.

## References

[1] Margerison JH , Corsellis JA . Epilepsy and the temporal lobes. A clinical, electroencephalographic and neuropathological study of the brain in epilepsy, with particular reference to the temporal lobes. Brain 1966;89:499–530. 5922048 10.1093/brain/89.3.499

[2] Duncan JS , Sagar HJ . Seizure characteristics, pathology, and outcome after temporal lobectomy. Neurology 1987;37:405–409. 3822133 10.1212/wnl.37.3.405

[3] Jackson GD , Berkovic SF , Duncan JS , et al. Optimizing the diagnosis of hippocampal sclerosis using MR imaging. AJNR Am J Neuroradiol 1993;14:753–762. 8517369 PMC8333381

[4] Woermann FG , Barker GJ , Birnie KD , et al. Regional changes in hippocampal T2 relaxation and volume: a quantitative magnetic resonance imaging study of hippocampal sclerosis. J Neurol Neurosurg Psychiatry 1998;65:656–664. 9810933 10.1136/jnnp.65.5.656 PMC2170343

[5] Jackson GD , Connelly A , Duncan JS , et al. Detection of hippocampal pathology in intractable partial epilepsy: increased sensitivity with quantitative magnetic resonance T2 relaxometry. Neurology 1993;43:1793–1799. 8414034 10.1212/wnl.43.9.1793

[6] Bernasconi A , Bernasconi N , Caramanos Z , et al. T2 relaxometry can lateralize mesial temporal lobe epilepsy in patients with normal MRI. NeuroImage 2000;12:739–746. 11112405 10.1006/nimg.2000.0724

[7] Coan AC , Kubota B , Bergo FP , et al. 3T MRI quantification of hippocampal volume and signal in mesial temporal lobe epilepsy improves detection of hippocampal sclerosis. AJNR Am J Neuroradiol 2014;35:77–83. 23868151 10.3174/ajnr.A3640 PMC7966486

[8] Van Paesschen W , Connelly A , King MD , et al. The spectrum of hippocampal sclerosis: a quantitative magnetic resonance imaging study. Ann Neurol 1997;41:41–51. 9005864 10.1002/ana.410410109

[9] Briellmann RS , Kalnins RM , Berkovic SF , et al. Hippocampal pathology in refractory temporal lobe epilepsy: T2‐weighted signal change reflects dentate gliosis. Neurology 2002;58:265–271. 11805255 10.1212/wnl.58.2.265

[10] Jack CR Jr , Sharbrough FW , Twomey CK , et al. Temporal lobe seizures: lateralization with MR volume measurements of the hippocampal formation. Radiology 1990;175:423–429. 2183282 10.1148/radiology.175.2.2183282

[11] Jack CR Jr , Sharbrough FW , Cascino GD , et al. Magnetic resonance image‐based hippocampal volumetry: correlation with outcome after temporal lobectomy. Ann Neurol 1992;31:138–146. 1575452 10.1002/ana.410310204

[12] Winston GP , Cardoso MJ , Williams EJ , et al. Automated hippocampal segmentation in patients with epilepsy: available free online. Epilepsia 2013;54:2166–2173. 24151901 10.1111/epi.12408 PMC3995014

[13] Meiners LC , van Gils A , Jansen GH , et al. Temporal lobe epilepsy: the various MR appearances of histologically proven mesial temporal sclerosis. AJNR Am J Neuroradiol 1994;15:1547–1555. 7985576 PMC8334415

[14] Duncan JS , Bartlett P , Barker GJ . Technique for measuring hippocampal T2 relaxation time. AJNR Am J Neuroradiol 1996;17:1805–1810. 8933861 PMC8337552

[15] Okujava M , Schulz R , Ebner A , et al. Measurement of temporal lobe T2 relaxation times using a routine diagnostic MR imaging protocol in epilepsy. Epilepsy Res 2002;48:131–142. 11823117 10.1016/s0920-1211(01)00325-4

[16] von Oertzen J , Urbach H , Blumcke I , et al. Time‐efficient T2 relaxometry of the entire hippocampus is feasible in temporal lobe epilepsy. Neurology 2002;58:257–264. 11805254 10.1212/wnl.58.2.257

[17] Bartlett PA , Symms MR , Free SL , et al. T2 relaxometry of the hippocampus at 3T. AJNR Am J Neuroradiol 2007;28:1095–1098. 17569966 10.3174/ajnr.A0505 PMC8134151

[18] Van Paesschen W , Sisodiya S , Connelly A , et al. Quantitative hippocampal MRI and intractable temporal lobe epilepsy. Neurology 1995;45:2233–2240. 8848199 10.1212/wnl.45.12.2233

[19] Namer IJ , Waydelich R , Armspach JP , et al. Contribution of T2 relaxation time mapping in the evaluation of cryptogenic temporal lobe epilepsy. NeuroImage 1998;7:304–313. 9626671 10.1006/nimg.1998.0331

[20] Lee DH , Gao FQ , Rogers JM , et al. MR in temporal lobe epilepsy: analysis with pathologic confirmation. AJNR Am J Neuroradiol 1998;19:19–27. 9432153 PMC8337339

[21] Coan AC , Kobayashi E , Li LM , et al. Quantification of hippocampal signal intensity in patients with mesial temporal lobe epilepsy. J Neuroimaging 2003;13:228–233. 12889169

[22] Coan AC , Bonilha L , Morgan PS , et al. T2‐weighted and T2 relaxometry images in patients with medial temporal lobe epilepsy. J Neuroimaging 2006;16:260–265. 16808828 10.1111/j.1552-6569.2006.00051.x

[23] Rodionov R , Bartlett PA , He C , et al. T2 mapping outperforms normalised FLAIR in identifying hippocampal sclerosis. Neuroimage Clin 2015;7:788–791. 25844331 10.1016/j.nicl.2015.03.004 PMC4375635

[24] Grunewald RA , Jackson GD , Connelly A , et al. MR detection of hippocampal disease in epilepsy: factors influencing T2 relaxation time. AJNR Am J Neuroradiol 1994;15:1149–1156. 8073986 PMC8333471

[25] Van Paesschen W , Revesz T , Duncan JS , et al. Quantitative neuropathology and quantitative magnetic resonance imaging of the hippocampus in temporal lobe epilepsy. Ann Neurol 1997;42:756–766. 9392575 10.1002/ana.410420512

[26] Sato S , Iwasaki M , Suzuki H , et al. T2 relaxometry improves detection of non‐sclerotic epileptogenic hippocampus. Epilepsy Res 2016;126:1–9. 27400070 10.1016/j.eplepsyres.2016.06.001

[27] Scott RC , Cross JH , Gadian DG , et al. Abnormalities in hippocampi remote from the seizure focus: a T2 relaxometry study. Brain 2003;126:1968–1974. 12805108 10.1093/brain/awg199

[28] Namer IJ , Bolo NR , Sellal F , et al. Combined measurements of hippocampal N‐acetyl‐aspartate and T2 relaxation time in the evaluation of mesial temporal lobe epilepsy: correlation with clinical severity and memory performances. Epilepsia 1999;40:1424–1432. 10528939 10.1111/j.1528-1157.1999.tb02015.x

[29] Coan AC , Appenzeller S , Bonilha L , et al. Seizure frequency and lateralization affect progression of atrophy in temporal lobe epilepsy. Neurology 2009;73:834–842. 19752449 10.1212/WNL.0b013e3181b783dd

[30] Townsend TN , Bernasconi N , Pike GB , et al. Quantitative analysis of temporal lobe white matter T2 relaxation time in temporal lobe epilepsy. NeuroImage 2004;23:318–324. 15325379 10.1016/j.neuroimage.2004.06.009

[31] Briellmann RS , Syngeniotis A , Fleming S , et al. Increased anterior temporal lobe T2 times in cases of hippocampal sclerosis: a multi‐echo T2 relaxometry study at 3 T. AJNR Am J Neuroradiol 2004;25:389–394. 15037460 PMC8158534

[32] Pell GS , Briellmann RS , Waites AB , et al. Voxel‐based relaxometry: a new approach for analysis of T2 relaxometry changes in epilepsy. NeuroImage 2004;21:707–713. 14980573 10.1016/j.neuroimage.2003.09.059

[33] Pell GS , Briellmann RS , Pardoe H , et al. Composite voxel‐based analysis of volume and T2 relaxometry in temporal lobe epilepsy. NeuroImage 2008;39:1151–1161. 18042496 10.1016/j.neuroimage.2007.09.061

[34] Kosior RK , Lauzon ML , Frayne R , et al. Single‐subject voxel‐based relaxometry for clinical assessment of temporal lobe epilepsy. Epilepsy Res 2009;86:23–31. 19464852 10.1016/j.eplepsyres.2009.04.001

